# Derivation of the Gait Deviation Index for Spinal Cord Injury

**DOI:** 10.3389/fbioe.2022.874074

**Published:** 2022-07-06

**Authors:** Diana Herrera-Valenzuela, Isabel Sinovas-Alonso, Juan C. Moreno, Ángel Gil-Agudo, Antonio J. del-Ama

**Affiliations:** ^1^ International Doctoral School, Rey Juan Carlos University, Madrid, Spain; ^2^ Biomechanics and Technical Aids Unit, National Hospital for Paraplegics, Toledo, Spain; ^3^ Neural Rehabilitation Group, Cajal Institute, CSIC–Spanish National Research Council, Madrid, Spain; ^4^ School of Science and Technology, Department of Applied Mathematics, Materials Science and Engineering and Electronic Technology, Rey Juan Carlos University, Madrid, Spain

**Keywords:** gait deviation index (GDI), spinal cord injury (SCI), gait impairment, three-dimensional (3D) kinematic gait data, walking index for spinal cord injury (WISCI), singular value decomposition

## Abstract

The Gait Deviation Index (GDI) is a dimensionless multivariate measure of overall gait pathology represented as a single score that indicates the gait deviation from a normal gait average. It is calculated using kinematic data recorded during a three-dimensional gait analysis and an orthonormal vectorial basis with 15 gait features that was originally obtained using singular value decomposition and feature analysis on a dataset of children with cerebral palsy. Ever since, it has been used as an outcome measure to study gait in several conditions, including spinal cord injury (SCI). Nevertheless, the validity of implementing the GDI in a population with SCI has not been studied yet. We investigate the application of these mathematical methods to derive a similar metric but with a dataset of adults with SCI (SCI-GDI). The new SCI-GDI is compared with the original GDI to evaluate their differences and assess the need for a specific GDI for SCI and with the WISCI II to evaluate its sensibility. Our findings show that a 21-feature basis is necessary to account for most of the variance in gait patterns in the SCI population and to provide high-quality reconstructions of the gait curves included in the dataset and in foreign data. Furthermore, using only the first 15 features of our SCI basis, the fidelity of the reconstructions obtained in our population is higher than that when using the basis of the original GDI. The results showed that the SCI-GDI discriminates most levels of the WISCI II scale, except for levels 12 and 18. Statistically significant differences were found between both indexes within each WISCI II level except for 12, 20, and the control group (*p* < 0.05). In all levels, the average GDI value was greater than the average SCI-GDI value, but the difference between both indexes is larger in data with greater impairment and it reduces progressively toward a normal gait pattern. In conclusion, the implementation of the original GDI in SCI may lead to overestimation of gait function, and our new SCI-GDI is more sensitive to larger gait impairment than the GDI. Further validation of the SCI-GDI with other scales validated in SCI is needed.

## 1 Introduction

Walking is an extraordinarily complex task requiring integration of the entire nervous system, making gait susceptible to a variety of underlying neurologic abnormalities, such as spinal cord injuries (SCIs). Incidence rates of SCI vary across countries between 10.4 and 83 new cases per million inhabitants per year (Wyndaele and Wyndaele, 2006), with a global prevalence between 236 and 1,009 per million (Cripps et al., 2011). From these, more than 95% experience mobility impairments resulting from the injury (Aspaym, Federación Nacional, 2012), which affects their quality of life. The average age when subjects experience an SCI is 33 years, and men are more affected than women with a 3.8:1 ratio (Wyndaele and Wyndaele, 2006). Therefore, although the incidence is considered low, the personal but also the social and economic consequences of spinal cord damage can be severe.

The overall objectives of rehabilitation in SCI are to increase personal independence and quality of life minimizing the socio-economic burden. Still, regardless of the severity of the SCI, the time after lesion or age at the time of injury, the restoration of walking is given high priority (Ditunno et al., 2008).Gait improvement in SCI following rehabilitation is assessed using different procedures, metrics, and tools: on the one hand, validated clinical tests on overall gait function, such as categorical and spatiotemporal-related walking and balance assessment measures such as the Walking Index for Spinal Cord Injury (WISCI) (Dittuno et al., 2001), the 10-meter walk test (10MWT) (Van Hedel et al., 2008), the timed up and go test (TUGT) (Podsiadlo and Richardson, 1991), the 6 min walking test (6MWT) (Brooks et al., 2003), and the Berg balance scale (BBS) (Berg, 1989) (Wyndaele and Wyndaele, 2006); on the other hand, tests of motor function and spasticity assessment, such as the lower extremity motor score (LEMS) and the modified Ashworth scale (MAS), respectively; and finally, instrumental techniques including dynamometry and three-dimensional gait analysis (3DGA). The latter is the most comprehensive and precise technology to analyze gait that allows to objectively assess lower limb kinematics and kinetics, thus providing a powerful tool for quantifying gait impairment and, therefore, to assist decision-making for clinicians (Patrick, 2003; Baker, 2006; Baker et al., 2016; Murphy et al., 2019; Sinovas-Alonso et al., 2021).

The main feature of 3DGA is that it provides a large amount of data describing the spatiotemporal gait parameters, together with three-dimensional (3D) pelvis, thigh, leg, and foot kinematics, as well as hip, knee, and ankle joint kinematics and kinetics during a gait cycle, along with specific values for each one of the gait phases and events (Gage, 1991). This extensive information, usually presented with many graphs and tables, is often both difficult and impractical to be understood by clinicians (Whittle, 1996; Patrick, 2003). Therefore, it is recognized that clinical interpretation of the 3DGA results needs to be facilitated to increase its usefulness in clinical settings. One way to achieve this goal is to develop and implement straightforward, easy to interpret metrics that merge data from 3DGA and yield a metric —or set of metrics— that describe overall gait deficits. One such metric is the Gait Deviation Index (GDI), which is a dimensionless multivariate measure of overall gait pathology represented as a single score that indicates the gait deviation from a normal gait pattern average (Schwartz and Rozumalski, 2008). It is calculated upon the kinematics of pelvis and hip in the three planes in space, knee, and ankle in the sagittal plane and foot progression angle.

Originally, a dataset with more than 6,000 strides of children with cerebral palsy (CP) was built to develop the GDI (Schwartz and Rozumalski, 2008). Based on these data, the authors derived a set of independent joint rotation patterns, referred to as gait features, so that, when combined linearly, high-quality reconstructions of gait curves can be obtained. In order to select the least amount of features needed to represent the whole CP gait profile dataset, they considered two criteria: 1) the set of features selected must account for at least 95% of the overall variance of the whole dataset, and 2) they must provide high-fidelity reconstructions of any gait curve with respect to the original curve. Applying these criteria, the authors found that 15 features out of 459 were enough to account for 98% of the total variance of the whole dataset and allowed to reconstruct the gait curves with 98% fidelity on average. These 15 features were organized into a matrix used as an orthonormal basis to calculate the representation of any gait curve. Afterward, to obtain the GDI, the Euclidean distance between this representation and the average of a set of control strides that may be introduced by the user, is calculated, representing the deviation of a gait pattern from a control group of typically developing (TD) children. Last, this value is scaled to improve the interpretability of the index, so that every 10 points of GDI below 100 correspond to one standard deviation away from the control pattern, whereas a score ≥100 represents a gait without any pathology (Schwartz and Rozumalski, 2008).

Ever since, that 15-feature basis originally developed from data of children with CP has been widely used to calculate the GDI across different conditions, including post-stroke hemiparetic gait (Correa et al., 2017; Guzik and Drużbicki, 2020), Duchenne muscular dystrophy (Sienko Thomas et al., 2010), Parkinson’s disease (Galli et al., 2012; Speciali et al., 2013), arthritis (Broström et al., 2013; Esbjörnsson et al., 2014; Kobsar et al., 2019; Bazarnik-Mucha et al., 2020), lower limb amputations (Eshraghi et al., 2014; Kark et al., 2016), degenerative spinal pathologies (Mar et al., 2019; Trivedi et al., 2021; Zhou et al., 2021), genetic disorders (Ito et al., 2020; Mindler et al., 2020), congenital disorders (Eriksson et al., 2015; Garman et al., 2019), the effect of the Covid-19 on physical function (Ito et al., 2021), and mostly in CP (Schwartz and Rozumalski, 2008; Molloy et al., 2010; Cimolin et al., 2011; Sagawa et al., 2013; Massaad et al., 2014; Wilson et al., 2015; Malt et al., 2016; Ito et al., 2019; Rasmussen et al., 2019).

The GDI has therefore become a clinically relevant score partly because it is easy to interpret and compute. Nevertheless, the basis provided in Schwartz and Rozumalski (2008) has proven to account for the variance in gait patterns and to reconstruct gait vectors with high fidelity, only in pediatric CP population. Significant differences in gait patterns among pediatric and adult population have been described (Cupp et al., 1999; Ganley and Powers, 2005; Bleyenheuft et al., 2012), as well as both clinical and biomechanical differences among the different neurological disorders. Furthermore, when applied only to CP, differences in GDI were found between adult and pediatric population (Maanum et al., 2012). Actually, the authors in the original work of the development of the index suggested that the methodology could be used in other sets of data (Schwartz and Rozumalski, 2008) but instead of developing a new basis for each condition, the articles found in the literature implement the GDI using the original basis regardless of the population. Therefore, straightforward application of the GDI derived in Schwartz and Rozumalski (2008) in other populations than pediatric CP can lead to a misleading interpretation of the gait data.

To date, no studies have attempted to validate the GDI in SCI. To our knowledge, only two articles have investigated its application under this condition (Hwang et al., 2021; Sinovas-Alonso et al., 2022). One of them uses the index to quantify and characterize gait patterns in ambulatory children and adolescents with transverse myelitis with respect to a normal gait pattern (Hwang et al., 2021). In this work, the difference in gait between patients and TD children was assessed with the GDI, without addressing the discriminative validity of the scale within different levels of impairment. The work presented in Sinovas-Alonso et al. (2022) compared the GDI and the WISCI II, showing limited discriminative properties of the GDI in SCI because there were statistically significant differences in the GDI values only between levels 13, 19, 20, and the control group. Therefore, the applicability of the GDI to SCI population that leads to discriminate the heterogeneity of gait impairment is still an open question calling for investigation.

The main objective of this article is to investigate the application of the mathematical methodology behind the GDI (Schwartz and Rozumalski, 2008) to a dataset of adults with SCI, resulting in the new SCI-GDI. Then, an evaluation of the differences between new SCI-GDI with the original GDI is presented, assessing the need for a specific GDI for SCI. Last, the relationship between our SCI-GDI and the WISCI II, the most validated scale in SCI developed specifically for this population (Sinovas-Alonso et al., 2021), is further presented to investigate the differences between the GDI and our novel SCI-GDI in terms of stratification and sensitivity to walking impairment with respect to a validated scale.

## 2 Materials and Methods

### 2.1 Participants

A dataset containing the kinematic data from 3D gait analysis of patients with SCI was used in this study. The 3DGA were conducted between August 2019 and July 2021 at the Biomechanics and Technical Aids Unit of the National Hospital for Paraplegics of Toledo, Spain. Patients aged ≥16 years old with diagnosis of SCI, regardless of the etiology, time since injury, injury level, or injury severity were included. A total of 302 strides from patients aged between 16 and 70 years old (33.91 ± 17.86), with injury levels between C1 and L5 and the ASIA impairment scale (AIS) C to D were gathered. The ratio of males to females of the dataset is 3.25:1. The detailed demographic and clinical characteristics of the sample are presented in Table 1. In addition, a control group with the 3D kinematic gait data of 446 strides from adults without gait pathologies was collected. These healthy volunteers (HV) were between 18 and 63 years old (35.10 ± 15.41), and the ratio of females to males was 1.63:1.

**TABLE 1:** Demographic and clinical characteristics of the samples in the train and validation datasets.

Characteristic	Type	Train (*n* = 302)	Validation (*n* = 72)
Age	16–25	156	52
	26–40	32	0
	41–60	79	10
	>60	35	10
AIS	A	0	10
	C	36	10
	D	256	36
	Cauda equina	10	10
	N.A. (congenital)	0	6
Time since injury	6 months (incl.) or less	58	10
	6 months (excl.) to 1 year (incl.)	40	0
	1 (excl.) to 5 years (incl.)	86	26
	More than 5 years	92	30
	Congenital	26	6
Injury level	C1–C8	153	0
	T1–T6	12	26
	T7–T12	68	20
	L1–L5	69	20
	N.A. (Congenital)	0	6
WISCI II level	12	2	0
	13	6	0
	15	18	10
	16	65	26
	18	12	6
	19	87	0
	20	112	30

All patients and HV signed an informed consent to perform the gait analysis. The study protocol was approved by the local bioethics committee (Clinical Research Ethics Committee at Complejo Hospitalario Universitario de Toledo, no. 823) and conformed to the Declaration of Helsinki.

### 2.2 Experimental Procedure

A Codamotion^®^ motion capture system (Charnwood Dynamics Ltd, United Kingdom) was used to capture 3D kinematic gait data. The standard protocol with 22 active markers placed on the lower limbs (Charnwood Dynamics Limited, 2004), three scanners, and two force platforms Kistler 9286A (Kistler Group, Switzerland) in the center of a 10-meter walkway were used. Post-processing was performed using the software ODIN v. 2.02 (Codamotion Ltd., England, United Kingdom). The subjects were asked to walk barefoot at a comfortable speed with the minimum external assistance required. Five complete gait cycles were recorded and time-normalized. For patients who were not able to complete five trials, at least three cycles were gathered.

### 2.3 Overview of the Calculation of the Gait Deviation Index

The GDI derivation procedure was described in detail in Schwartz and Rozumalski (2008). The calculation is based on a matrix with kinematic data from several walking strides where each column vector is a stride represented by nine joint angles of a whole gait cycle extracted at 2% increments: three planes for the pelvis and hip, knee flex/extension, ankle dorsi/plantarflexion, and foot progression angle. Singular value decomposition (SVD) of the matrix is computed to obtain its singular values and singular vectors. Using the latter, referred herein as gait features, the authors build an orthonormal basis that is both optimal to maximize the variance accounted for (VAF) of the whole dataset, and useful to reconstruct gait data. When multiplying the first *m*-vectors of this basis by any gait vector, an *m* ^*th*^ order approximation of the vector is obtained, therefore forming a vectorial basis. The accuracy of this reconstruction is calculated with its projection onto the original vector, normalized by the original gait curve. Two criteria were used to find out the minimum *m* features needed to form a reduced order basis such that it represented the whole CP dataset; first, these first *m* features accounted for 98% of the total variance of the original dataset, and second, the accuracy of the *m* ^th^ order reconstructed curves was 98% on average. The authors in Schwartz and Rozumalski (2008) found that 15 features were sufficient to form the reduced order basis. Last, using the approximation of a gait vector obtained with this basis, its Euclidean distance with an average gait vector from a control group is calculated and scaled to obtain the GDI.

### 2.4 Data Analysis

An overview of the data analysis performed is presented in this section. The detailed description of each step is found on the following subsections. Henceforth, all data analysis was performed with Matlab R2019a (The MathWorks, Inc., Natick, MA, United States).

**FIGURE 1:**
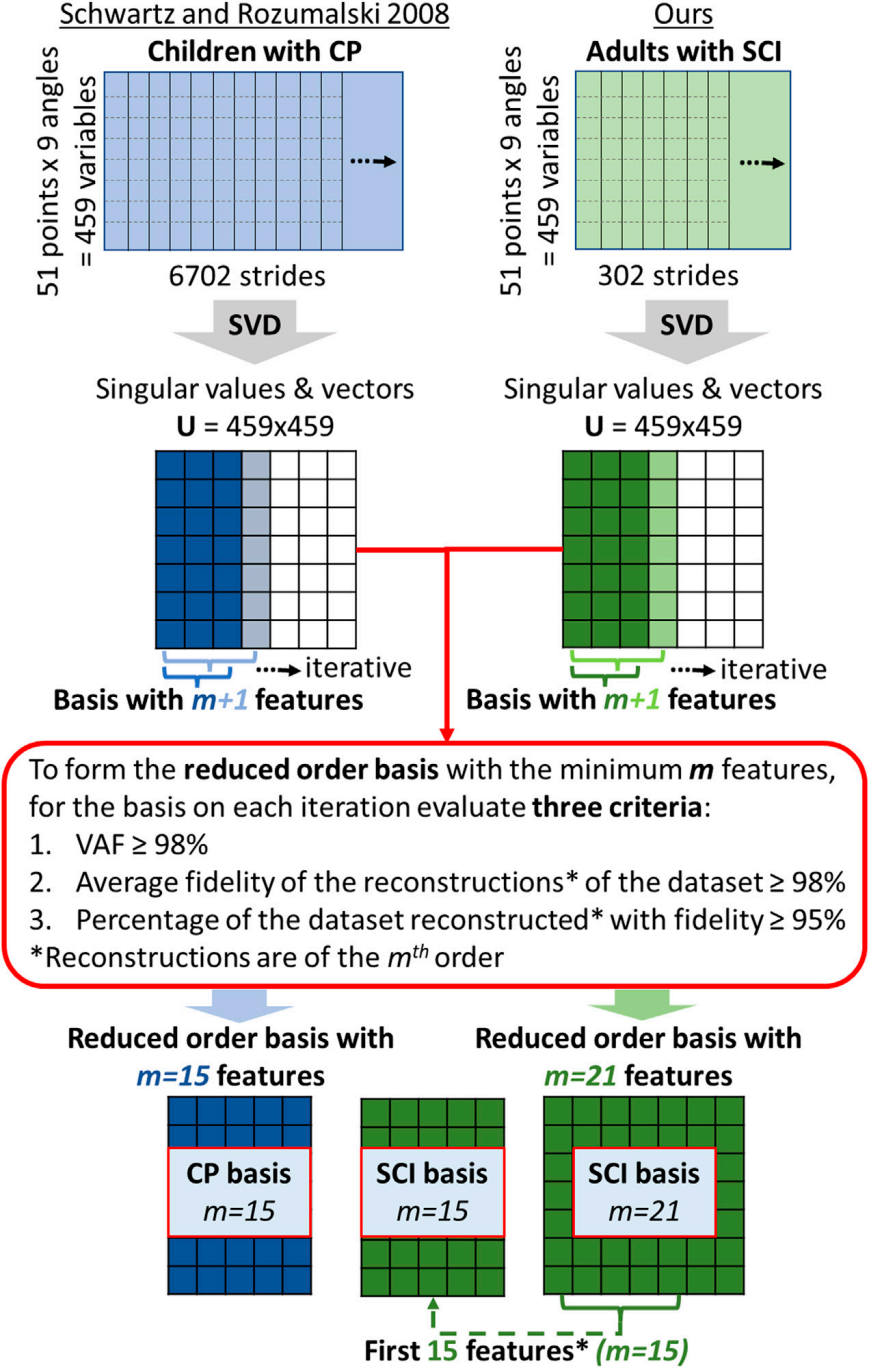
Diagram showing the steps followed to obtain the three reduced order bases compared in this article. The column on the left, with matrices in blue, represents the process followed in the original article by Schwartz and Rozumalski (2008), whereas the green matrices on the right correspond to the steps performed in this work, using SCI gait data. Note that the reduced order SCI basis with 15 features, located in the middle at the bottom of the diagram is merely the set of the first 15 features of the 21-feature reduced order SCI basis. We compare the results of the three criteria in red when using this basis because the number of features in the basis determines the order of the reconstructions of the gait curves. Thus, it is fair to compare the quality of the reconstructions of the same order.

Using the dataset described previously, the first step of the data analysis was the computation of what we call the SCI-GDI basis, that is, the optimal reduced order orthonormal basis to reconstruct gait data of SCI with high fidelity and to account for most of the variance of the SCI dataset (data analysis details in Section 2.4.1). Once the SCI basis is formed with the sufficient amount of gait features *m*, in order to assess the appropriateness of computing the GDI in adult population with SCI using the original GDI basis, developed using a dataset of pediatric patients with CP (Schwartz and Rozumalski, 2008), we compared the quality of the reconstructions of the whole SCI dataset obtained with three bases: our SCI-GDI basis, the original GDI basis comprises 15 gait features (Schwartz and Rozumalski, 2008), and the first 15 features of our SCI-GDI basis. From the three bases, the latter was used to compare the fidelity of the reconstructions of the same order as those obtained with the original GDI basis. Figure 1 shows a diagram of the steps followed to obtain these three bases. In addition, to assess the generalizability of the new SCI basis in foreign data, we computed the quality of reconstruction in a set of strides not used during the computation of the SCI basis.

Afterward, we calculated the SCI-GDI of the dataset using our basis and compared it with the WISCI II scale, to assess the stratification of gait impairment and discriminative properties of the new index (data analysis details in Section 2.4.2). To our knowledge, there is only one work published that studies the relationship of the GDI with a scale developed specifically for SCI and validated in this population, which is the WISCI II (Sinovas-Alonso et al., 2022). We did not perform comparisons with other validated metrics in SCI, such as 10MWT, 6MWT, TUGT, or the BBS because these were not available. Last, we compared and correlated the GDI with the SCI-GDI in our dataset to find out whether there is an actual difference between both indexes when computed in the same set of SCI subjects, in order to recommend one index over the other in this specific population of adults with SCI (data analysis details in Section 2.4.3).

#### 2.4.1 Computation of the Spinal Cord Injury-Gait Deviation Index Basis

In this work, 302 strides from SCI patients were used to form a matrix to compute the reduced order optimal basis. We refer to this data as our train dataset. We performed a grid search considering values of *m* between 10 and 30 to find the minimum features needed to form the optimal reduced order SCI basis with the two criteria explained at the end of Section 2.3. We also considered the percentage of gait vectors of the whole dataset reconstructed with a fidelity ≥95%, a parameter reported in the original work of the derivation of the GDI (Schwartz and Rozumalski, 2008). Although our dataset could be considered small to perform Feature Analysis, especially when compared to the 6000-stride dataset of the original GDI work, it is possible to obtain reliable, high-quality solutions with small datasets if the communalities between the features are high because accurate recovery of population structure may be obtained with a small sample; thus, the size of the dataset will have little impact on the quality of the result (MacCallum et al., 1999). Communality is related to the VAF criteria previously defined in this work because it is defined as the proportion of the variance of the variable that is accounted for by the features (Hogarty et al., 2005). Therefore, to consider the validity of our dataset in this task, we performed a Monte Carlo cross-validation with 10 iterations to assess the stability of the result. On each iteration, five percent of the data was randomly removed before computing the SVD and a surrogate model was built. With each model, the three criteria were assessed to find the minimum *m* features that allowed fulfilling each criterion: VAF≥98%, average fidelity of the reconstructions ≥98%, and percentage of the dataset reconstructed with fidelity ≥95%. Small differences between the *m* values found on each run indicated similarity between the models and stable results (MacCallum et al., 1999).

Moreover, we compared these results with the quality of the 15th order reconstructions of all the gait vectors in the dataset using the basis provided in Schwartz and Rozumalski (2008), built with 15 features from CP patients, and also with the reconstructions obtained with the first 15 features of the basis calculated with our SCI dataset. Furthermore, to validate the generalizability of the new basis built from SCI gait data, a validation set was built with 72 additional strides that were not used to calculate the basis. These were reconstructed and compared using the three bases, and the reconstruction fidelity was assessed with the same criteria used in the train set, allowing to compare the quality of the reconstructions in foreign data.

#### 2.4.2 Comparison Between the Spinal Cord Injury-Gait Deviation Index and the Walking Index for Spinal Cord Injury II Scale

The SCI-GDI was calculated for each stride of both patients and HV using the reduced order orthonormal basis built in this work. Control group data, used as the reference gait pattern to compute the gait deviation, was collected at National Hospital for Paraplegics, as described in Section 2.1, following the same procedure used with the patients. Each gait analysis study had an associated WISCI II level, according to the walking impairment of the patient when recording the study. SCI-GDI data were grouped according to the corresponding WISCI II level, and HV data were considered as an additional set. The dataset included WISCI II levels 12, 13, 15, 16, 18, 19, and 20. Normal distribution for each group was assessed with Kolmogorov–Smirnov tests (*p* < 0.05).

To facilitate the analysis, a histogram of the SCI-GDI data comprised within each WISCI II level was calculated with a normal distribution curve fitted to its mean and standard deviation. A stratified result of the histograms was expected, in accordance with the ordinal nature of the WISCI II scale. Afterward, one-way ANOVA tests were performed between the SCI-GDI values of each pair of WISCI II levels to identify differences among groups (*p* < 0.05). In addition, a Kendall’s Tau-b correlation was run between both scales to assess their relationship.

#### 2.4.3 Comparison and Correlation Between the Spinal Cord Injury-Gait Deviation Index and the Gait Deviation Index

To seek differences between the original GDI, calculated from a basis derived from a CP pediatric population (Schwartz and Rozumalski, 2008), and the SCI-GDI, both indexes were calculated for each stride of the dataset using the HV data gathered in our institution. The results were grouped according to the WISCI II level of the sample. Normal distribution for each group was assessed with Kolmogorov–Smirnov tests (*p* < 0.05). Consequently, one-way ANOVA tests were performed between each pair of equivalent WISCI II levels to identify differences among groups (*p* < 0.05). In addition, to study the relationship between both indexes, Pearson’s correlation and linear regression were calculated between both GDI values using the whole dataset.

## 3 Results

### 3.1 Computation of the Spinal Cord Injury-Gait Deviation Index Basis

The Monte Carlo cross-validation demonstrated stable results in terms of differences no larger than one in the minimum number of features necessary to build the basis, according to the criteria defined. On average, 19.3 ± 0.5 features were sufficient to account for 98% of variance of the dataset. Nevertheless, 21.0 ± 0.0 features were necessary to reconstruct the vectors of the dataset with an average fidelity of 98%. At *m* = 21, 97.9 ± 0.4% of the whole dataset was reconstructed with a fidelity of at least 95%. Therefore, *m* = 21 was set as the minimum number of features to build the basis to represent the whole SCI gait dataset.

**FIGURE 2:**
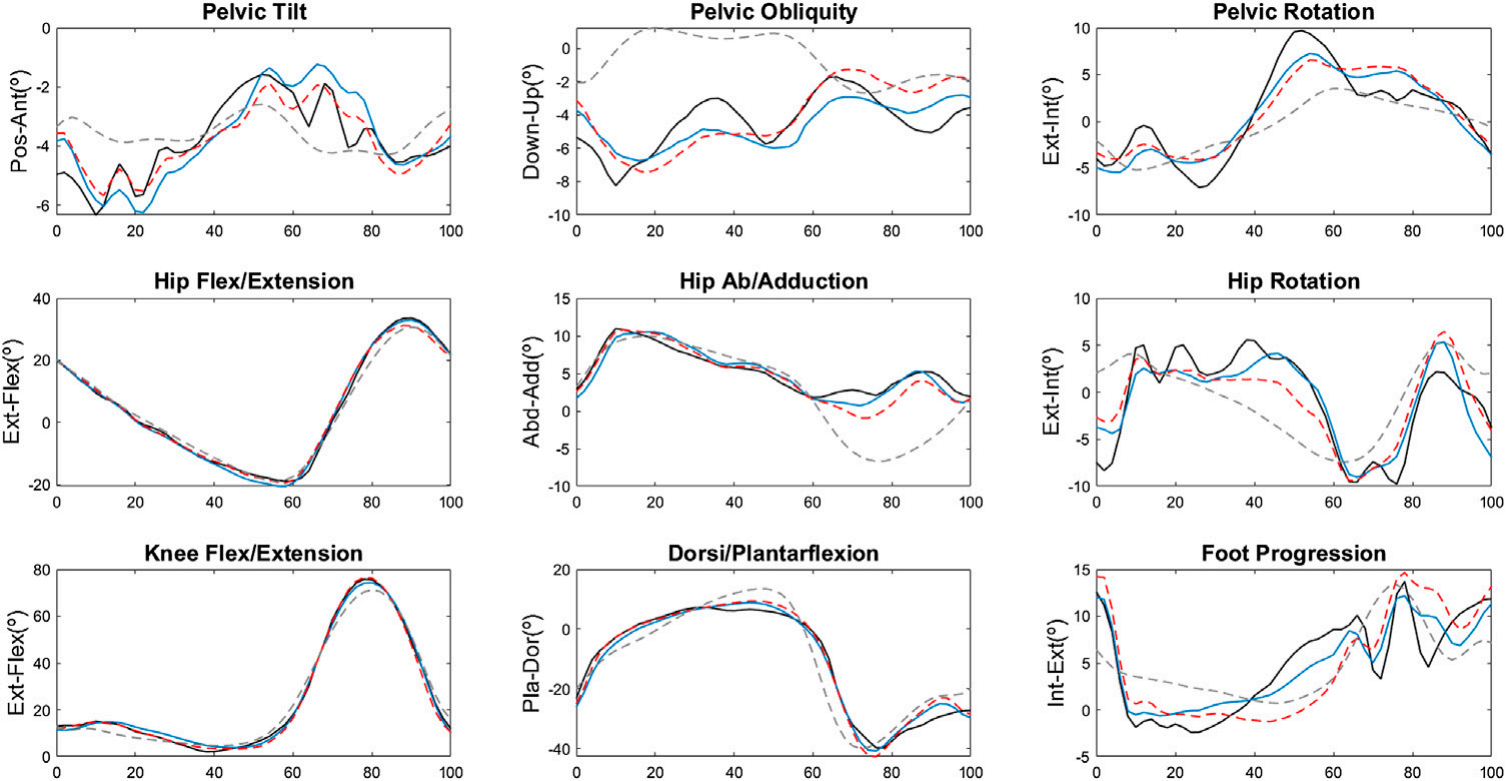
Kinematic reconstructions of a validation stride using the three bases. The black line is the original curve, the blue line is the result when using the SCI basis with *m* = 21, the red dashed line corresponds to the reconstruction with the SCI basis with *m* = 15, and the gray dashed line is the reconstruction with the CP basis (Schwartz and Rozumalski 2008). For all nine angles, the reconstructions with the original CP basis provide the largest deviation from the original curve.

The comparison of the quality of reconstruction of the whole dataset when using our SCI basis with *m* = 21, our SCI basis with *m* = 15 and the basis of the original GDI derived for children with CP with *m* = 15 is presented in Table 2. The best results in terms of average fidelity of the reconstructions and percentage of vectors reconstructed with a fidelity ≥95% were obtained with our *m* = 21 basis, followed by our basis built using only the first 15 features. Less than 50% of the dataset was reconstructed with a quality of at least 95% when using the basis provided in Schwartz and Rozumalski (2008). Note that it was not possible to calculate the VAF with the original GDI basis because the singular values of the original dataset are not publicly available. In the validation set, the results for all criteria followed the same pattern when using each type of basis but all scores were lower than those obtained in the train dataset. In Figure 2, we present the reconstructions obtained with the three bases on a sample of the validation dataset with a SCI-GDI of 55.59 and a GDI of 60.03.

**TABLE 2:** Comparison of the quality of reconstruction of the whole dataset when using our SCI basis with *m* = 21, our SCI basis with *m* = 15 and the basis of the original GDI derived for children with CP with *m* = 15 (Schwartz and Rozumalski 2008).The best results in terms of average fidelity of the reconstructions and percentage of vectors reconstructed with a fidelity ≥95% are obtained with our m = 21 basis, followed by our basis built using only the first 15 features.

Basis (n^o^ of features)	Set	VAF	Average fidelity of reconstruction	% of gait vectors reconstructed with average fidelity ≥95 (%)
SCI basis (*m* = 21)	Train	98.27%	97.99% ± 1.54%	97.86
	Validation		94.74% ± 4.88%	72.22
SCI basis (*m* = 15)	Train	97.11%	96.58% ± 2.49%	83.11
	Validation		92.40% ± 6.64%	52.78
CP basis (*m* = 15) (10)	Train	N/A	93.13% ± 5.51%	44.70
	Validation		90.73% ± 7.81%	40.28

VAF, variance accounted for; SCI, spinal cord injury; CP, cerebral palsy; N/A, not applicable.

### 3.2 Comparison Between the Spinal Cord Injury-Gait Deviation Index and the Walking Index for Spinal Cord Injury II Scale

**FIGURE 3:**
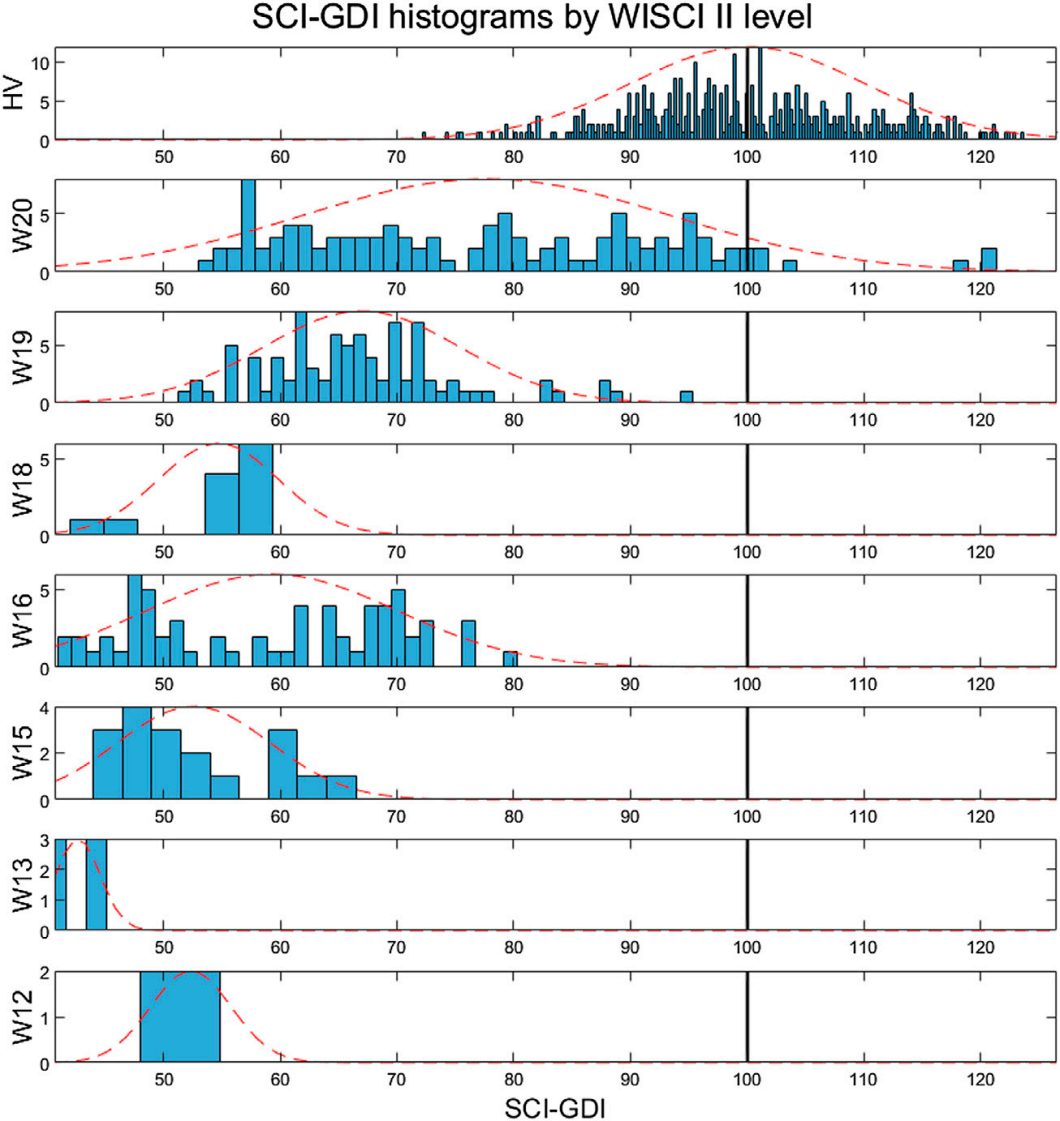
Histograms of the SCI-GDI stratified by the WISCI II level (12–20 and control). The dotted line represents the normal distribution curve fitted to the data within each level. The vertical black line indicates the control mean.

The results showed that the SCI-GDI is normally distributed across all WISCI II levels and in the HV group. Table 3 presents the distribution of the data, the mean and the standard deviation of the SCI-GDI values comprised in each WISCI II level. There is a trend of increasing SCI-GDI with a decreasing level of functional limitation in WISCI II levels 13 to 20 and in the control group, except in level 18, with an average SCI-GDI lower than the average on level 16. This can be easily seen in Figure 3 that shows the histograms of the SCI-GDI stratified by WISCI II level. Statistically significant differences were found between control group, levels 13, 19 and 20 with all other groups, and additionally, between levels 15 and 16. In essence, all the levels had statistically significant differences except from 12 to 18 (see Table 3). Furthermore, both SCI-GDI and WISCI II have a strong, positive correlation of 0.460, which is statistically significant, according to Kendall’s coefficient of rank correlation (*p* = 1.63e-26) (Botsch, 2011).

**TABLE 3:** Descriptive statistics of the SCI-GDI values within each WISCI II level. Numbers in parentheses indicate statistically significant differences found with an ANOVA (*p* < 0.05). The values marked with * indicate statistically significant differences found only in the SCI-GDI but not with the original GDI (Sinovas-Alonso, Herrera-Valenzuela, et al., 2022).

WISCI II	No. strides	Mean ± S.D. SCI-GDI	Minimum SCI-GDI	Maximum SCI-GDI	Normally distributed (K–S test)
C _(12, 13, 15, 16, 18, 19, 20)_	446	100.0 ± 10.0	72.2	126.5	True
20 _(12, 13, 15, 16, 18, 19, C)_	112	77.7 ± 15.8	53.8	120.8	True
19 _(12, 13, 15, 16, 18, 20, C)_	87	67.0 ± 8.4	51.7	95.0	True
18 _(13, 19, 20, C)_	12	54.7 ± 5.1	42.0	59.2	True
16 _(13, 15*, 19, 20, C)_	65	59.3 ± 10.8	41.0	80.2	True
15 _(13, 16*, 19, 20, C)_	18	52.6 ± 6.6	44.8	66.2	True
13 _(12, 15, 16, 18, 19, 20, C)_	6	42.7 ± 1.9	40.7	44.9	True
12 _(13, 19, 20, C)_	2	52.4 ± 3.5	49.9	54.8	True

WISCI II, Walking Index for Spinal Cord Injury II; SCI-GDI, gait deviation index for spinal cord injury; S.D., standard deviation; C, control; K–S Test, Kolmogórov–Smirnov test.

### 3.3 Comparison and Correlation Between the Spinal Cord Injury-Gait Deviation Index and the Gait Deviation Index

**FIGURE 4:**
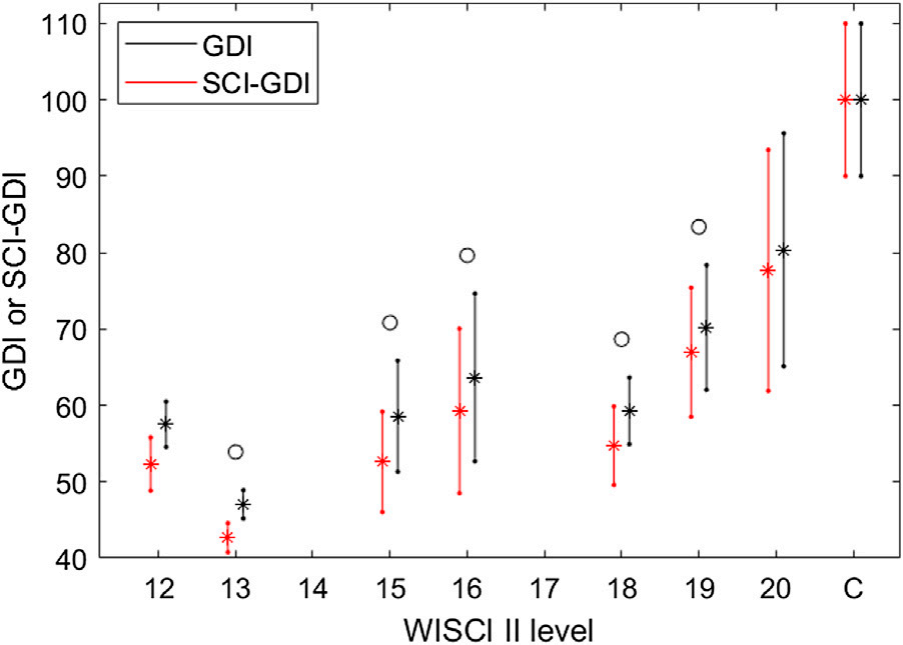
Average ± one standard deviation for GDI (black) and SCI-GDI (red) for each WISCI II level. In all levels, GDI values are greater than SCI-GDI values. WISCI II levels with a statistically significant difference between both indexes are marked with a circle.

**FIGURE 5:**
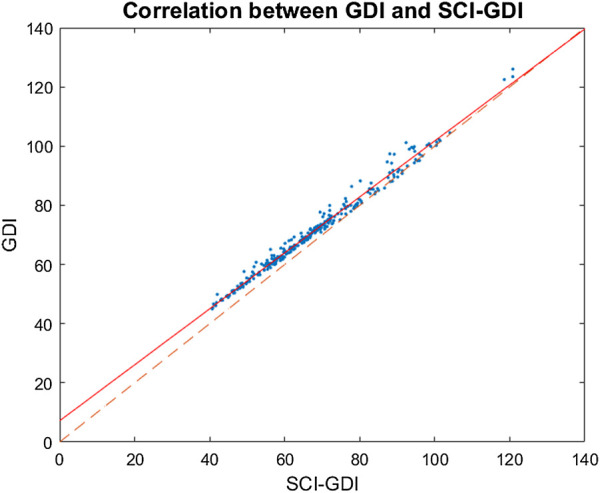
Strong linear correlation between GDI and SCI-GDI was found (*r* = 0.993). The linear regression between both indexes, represented by the continuous line, is given by the equation SCI_GDI=1.0573∗GDI−7.5915 . The dashed line indicates the 1:1 axis. For all the samples, GDI values are larger than SCI-GDI values. The difference between both indexes is larger in data with greater impairment and it reduces progressively toward a normal gait pattern.

Both SCI-GDI and GDI are normally distributed across all WISCI II levels and in the control group, according to the KS tests. When comparing the GDI and SCI-GDI values within each WISCI II level (Figure 4), statistically significant differences were found between all levels except for 12, 20, and the control group. For all levels, average GDI was greater than average SCI-GDI and followed the same pattern among adjacent WISCI levels (Figure 4). Furthermore, a strong linear correlation between both GDI and SCI-GDI was found (*r* = 0.993) (Figure 5), although both deviate at lower values.

## 4 Discussion

The main objective of this article was to derive a specific GDI applicable to SCI (SCI-GDI). Our hypothesis was that, since the GDI was obtained from a database of children with CP, the application to SCI would not correctly represent the gait impairments of this population. To this extent, we derived a GDI following the methodology originally proposed (Schwartz and Rozumalski, 2008) to a gait database of adults with SCI to obtain the SCI-GDI, studied the correlation of our SCI-GDI with the WISCI II, and compared the GDI and the SCI-GDI to assess their differences.

Although our dataset to compute the reduced order SCI basis contained fewer number of steps than the original one, there is no rule of thumb to define the minimum size that a dataset should have to perform SVD and feature selection given an initial number of features (Bandalos and Boehm-Kaufman, 2009). Different recommendations stated in the literature and some studies demonstrate that it is feasible to obtain quality solutions with small datasets if certain conditions are met, like having data with high communalities (MacCallum et al., 1999; Hogarty et al., 2005; Mundfrom et al., 2005). During the process of finding the number of gait features necessary to form our optimal reduced order SCI basis, a high variables-to-factors ratio and stable results with variations of at most one feature in the Monte Carlo cross-validation suggested that our dataset is large enough to represent robustly the variety of gait patterns within the population of SCI comprised in the data, by using linear combinations of the information. Nevertheless, a larger dataset would be recommendable, given the number of features of the original matrix in which SVD is performed.

Regarding the process of defining the minimum number of features to form the reduced order basis, our results showed that *m* = 19 was enough to account for at least 98% of the variance comprised in the dataset, indicating high communalities in the data, and suggesting that the size of our dataset is acceptable to be used in this study. Two more features are necessary (*m* = 21) to reconstruct the curves within the dataset with an average fidelity of 98%. This difference is understandable because the first criterion was calculated with the singular values of the PCA while the second one depended on the singular vectors. In addition, at *m* = 21, almost 98% of the whole dataset was reconstructed with a fidelity of at least 95%, only 1% less than the results presented by Schwartz and Rozumalski (2008) in the original derivation of the GDI in CP. Therefore, we defined *m* = 21 to build our SCI basis because both criteria must be fulfilled in order to build a basis that represents the whole dataset. It is important to note that these results indicate that six features or more are necessary to represent the variety of gait in SCI when compared to the 15 features that were sufficient in CP (Schwartz and Rozumalski, 2008). These results suggest a larger variance in the kinematics of gait in SCI when compared to CP, which may be related to the heterogeneity of the clinical forms of incomplete SCI depending on the level of injury and AIS. Hence, the original 15-feature basis of the GDI may not account for the variety or reconstruct with enough precision gait vectors in SCI.

In this regard, the results presented in Table 2 show that when calculating the quality of the reconstructions obtained with the original basis of the GDI (Schwartz and Rozumalski, 2008) on our train dataset, fidelity drops from an “ideal” value reported in Schwartz and Rozumalski (2008) of 98–93% and most importantly, only 44.70% of the dataset is reconstructed with a fidelity of at least 95%. These findings support the fact that the implementation of the GDI in SCI is not recommended because the dataset used to derive the GDI basis was a pediatric CP population and there are differences in the etiology of the neurological impairment, clinical consequences related to function and maturity of gait between adults and children that cause differences in gait patterns among populations. Indeed, even when using only the first 15 features of our SCI basis, the average fidelity of reconstruction is 1.41% lower when compared to our SCI basis with 21 features, but only 83.11% of the dataset is reconstructed with high quality, which is almost twice the value obtained with the CP basis. This means that even when using a basis built with data of adults with SCI, 15 features are not enough to represent and reconstruct with accuracy the whole dataset, but are better than using the 15 features from the original CP basis.

The results obtained using the validation dataset follow the same pattern, supporting the previous findings and indicating that results are not due to an overfitting to our dataset. Nevertheless, we highlight the fact that all the values obtained when using the validation dataset are lower than the corresponding results in the train dataset, suggesting that using more train data would be recommended to obtain a SCI basis that provides more generalizable results, as reflected by smaller differences in performance when evaluating the criteria in both sets.

Moreover, the reconstruction of a single sample of the validation set with a large level of gait deviation (WISCI II = 18, SCI-GDI = 53.47, and GDI = 60.43) in Figure 2 shows that the reconstructions obtained with the CP basis are poorly related to the original vector, whereas reconstructions with the SCI basis with 15 features have a better quality and the most accurate results are obtained with our 21st order reconstructions. It is also noteworthy that pelvic movement in the three axes is poorly reconstructed in all cases. We hypothesize this might be because the pelvis is the most complicated segment to model accurately and with reliability during a 3DGA (O'Sullivan et al., 2010). The anatomical landmarks used to place or align the pelvic markers on most motion capture systems, including the Codamotion, are the anterior and posterior superior iliac spines. These are bony protuberances in the pelvis covered with adipose tissue; therefore, the markers cannot be placed accurately on the subjects (C-Motion Wiki Documentation, 2019) and are prone to soft tissue artefacts (Langley et al., 2019). Based on these markers, the position of the pelvis is estimated; thus, the sources of error propagate from marker positioning to the computation of the kinematics of the segment. The improvement of the register of the pelvis during 3DGA is out of the scope of this article, but the issues for precisely estimating the position of the pelvis during a 3DGA are common to any capture, and therefore it is a limitation present in any 3DGA, and not only applicable to the calculation of the GDI or the SCI-GDI. Although it is not stated in the work by Schwartz and Rozumalski (2008), in their Figure 2 it seems they identified similar difficulties in achieving precise pelvic representations. On the contrary, kinematics in the sagittal plane for the knee, hip, and ankle have more precise reconstructions in the three examples. The angles that are better reconstructed might be more useful in attempts to derive indexes that use less variables than the nine used in the GDI.

In other respects, the comparison between the SCI-GDI and the WISCI II scale showed the stratification expected in levels 13 to 20 and in the control group, except in level 18 (see Figure 3), similarly to the results obtained when using the GDI (Sinovas-Alonso et al., 2022). Nevertheless, an important difference is that only in the SCI-GDI, levels 15 and 16 showed a statistical difference, unlike in GDI (see Table 3). Therefore, the SCI-GDI provides a better discrimination of more WISCI II levels when compared to the GDI. The new index managed to discriminate all the levels comprised in the dataset except for 12 and 18, which have few data, especially level 12. Therefore, the SCI-GDI provides a good discrimination of most WISCI II levels between 13 and 20. We hypothesize that level 18 is hard to discriminate first because there are few data in this level, and second because it indicates the use of braces to improve functionality, which blocks differently hip, knee, and ankle joints, depending on the nature of the orthosis, imposing a less-physiological gait pattern. Asking a patient who usually uses braces to walk without them, even in short distances like during a 3DGA, increases considerably the difficulty of the task, highlighting the impairment of gait with respect to the normal pattern. That might be why impairment as measured by the GDI is increased in level 18 with respect to level 16, and that does not include the use of braces but the use of crutches that affect mostly gait kinetics instead of kinematics. Moreover, the strong, positive correlation found between both scales (τ_B_ = 0.460) show that they are related and measure gait impairment while at the same time, with a τ_B_ value far from a perfect correlation, representing different aspects of gait pathology. Furthermore, the comparison between the GDI and SCI-GDI demonstrated that both indexes are statistically different (see Figure 4) for all the WISCI II levels analyzed that include any type of walking assistance, supporting the importance of using a gait deviation index derived from a proper sample, in essence, a SCI adult population. Level 12 is not analyzed in detail because it is poorly represented with only two samples.

The results shown in Figure 4 indicate that walking impairment is less penalized by the GDI when compared to the SCI-GDI. This is congruent with the findings stated previously in our work because if the CP basis covers a smaller variance on gait patterns and provides low quality reconstructions on SCI, the GDI calculated from a SCI gait vector using this basis might be based on a poorer representation of the original SCI vector and therefore, less penalized than when the vector is better reconstructed and includes the alterations present in the gait curves. Furthermore, the similar patterns between both deviation indexes across all WISCI II levels presented in Figure 4 and the strong linear correlation (*r* = 0.993) support that our SCI-GDI represents the same aspects of gait impairment as the GDI. The linear relationship between both indexes presented in Figure 5 show clearly that in higher values of GDI, the differences between GDI and SCI-GDI reduce. Thus, the application of the GDI in SCI could provide misleading information about the dimension of the gait impairment, especially in patients with greater neurological damage. Therefore, our SCI-GDI is more sensitive to larger gait impairment than the GDI, but the difference between both indexes reduces progressively towards normal gait. These findings are congruent with the statistical differences found between both indexes for all WISCI II levels except for level 20 (see Figure 4), corresponding to individuals that do not require any assistance to walk. This makes sense because no difference in the degree of gait impairment is identified in subjects that do not need assistance to walk.

Our study had several limitations. First, as mentioned before, even though the computation of the SCI-GDI basis showed stable results, using a larger dataset would allow us to verify that our results (number of features *m*, VAF and reconstruction percentages) indeed remain independently of the number of strides in the database. Other limitations inherent to the SCI pathology is that due to the high variability of gait impairment in SCI ,which depends on several factors such as the neurological level of injury (NLI), the severity of the injury according to the AIS and the time since onset of injury, there is no topographic classification of SCI to assess an ordinal level of gait impairment, unlike other neurological pathologies such as CP (Gage, 1991). Therefore, it is not possible to compare or validate the SCI-GDI with neither the AIS nor the NLI. Even though we only compared the SCI-GDI with the WISCI II due to data availability, a more balanced distribution of the data within the WISCI II levels was desirable. In this regard, our study lacks data of other gait tests or scales validated in SCI, like the 6MWT, TUG, 10MWT, or BBS, to further validate the SCI-GDI. Such validation will also reinforce the need of developing specific GDIs for each condition instead of implementing the pediatric CP-based GDI to several populations without sufficient validation. Centers with gait datasets from other pathologies than CP (Schwartz and Rozumalski, 2008) and SCI (this work) can reproduce this methodology to develop specific gait deviation indexes for their specific pathologies.

In spite of these limitations, the SCI-GDI can be applied in any person with a SCI regardless of the severity or neurological level of injury, from 16 to 70 years old in both men and women. The most important changes in gait kinematics occur during adolescence, and gait is considered mature and steady afterward, with few changes (Cupp et al., 1999). Children have different gait kinematics than adults (Ganley and Powers, 2005) that are constantly changing through ages, and in elderly, around the age of 60–70, significant changes in gait are also reported (Prince et al., 1997). In addition, after an SCI is chronic, changes in gait are reduced mostly to those related to rehabilitation outcomes and are covered by the data included in our dataset. Likewise, the small differences in gait kinematics between men and women that are mostly present in the frontal plane of the pelvis and hip (Bruening et al., 2015) are not as conditioning as the gait limitations after an SCI, allowing the application of the SCI-GDI regardless of sex. We intentionally captured a wide variety of gait data of SCI with different severity, neurological level of injury, time since injury onset, sex, and age, to capture the largest variety in gait patterns we had access to, and guarantee that the SCI-GDI could properly represent any of these patterns. This leads us to suggest the implementation of the SCI-GDI in adults with SCI from 16 to 70 years old, using the electronic addendum provided in the Supplementary Material.

## 5 Conclusion

The SCI-GDI is calculated using a 21-feature vectorial basis derived from gait data of adult population with SCI, instead of the 15-feature basis used for the original GDI. Our index has better discriminative properties of more WISCI II levels than the original GDI when applied to adults with SCI and conforms to the stratification of gait impairment of the WISCI II scale. In addition, the SCI-GDI is more sensitive to larger gait impairment than the GDI, but its sensitivity decreases with less impaired gait function. Indeed, the implementation of the original GDI in SCI may lead to overestimation of gait function. The SCI basis also allows building higher-quality reconstructions of gait curves when compared to the original GDI basis. Although further validation of the index with other scales used in SCI would be of interest, we recommend its implementation in adults with SCI. It can be easily computed using the electronic addendum provided in the Supplementary Material.

## Data Availability

An electronic addendum is provided in the Supplementary Material to implement the SCI-GDI. Detailed instructions to compute the index are provided in the same file. The novelty of this article with respect to the original development of the GDI (Schwartz and Rozumalski, 2008) is the 21st feature SCI basis calculated. Therefore, the addendum provided has the same structure that the one provided to compute the GDI, but with our SCI basis.
